# Superfine grinding on the physicochemical properties, volatile compounds, and bioactive properties of *Forsythia suspensa* (Thunb.) Vahl fruit powder

**DOI:** 10.1016/j.fochx.2026.104018

**Published:** 2026-05-22

**Authors:** Yu Li, Tianjian Guo, Ruixi Gao, Yupeng Liu, Jun Li, Xiao Chen, Ghulam Murtaza, Han Cheng, Xianju Huang

**Affiliations:** a School of Pharmaceutical Sciences, South-Central Minzu University, Wuhan 430079, China; b Hubei Yaosheng Traditional Chinese Medicine Technology Co. Ltd, Zaoyang 441200, China; c Department of Pharmacy, COMSATS University Islamabad, Lahore Campus, Lahore 54000, Pakistan

**Keywords:** *Forsythia suspensa*, Superfine grinding, Physicochemical properties, Volatile compounds, Bioactive properties

## Abstract

In this study, *Forsythia suspensa* fruit was processed by traditional and superfine grinding, sieving to obtain four powders (FS50, FS100, FS150, FS200). The average particle size decreased from 185.33 μm to 27.93 μm, accompanied by a 79.15% increase in cell wall breakage rate, a 63-fold increase in specific surface area, enhanced total pore volume, greater exposure of hydrophilic groups, and improved hydration properties. Total flavonoids, forsythoside A, and phillyrin peaked in FS150. Significant differences were observed in the volatile compounds among the four powders, with 19 shared key compounds contributing prominently to the aroma. Bioactivity assays demonstrated that FS150 exhibited the strongest anti-inflammatory effects and DPPH/ABTS radical scavenging activities. Correlation analysis suggested close relationships among physicochemical properties and indicated that active components underlie anti-inflammatory and antioxidant activities. Overall, *Forsythia suspensa* fruit ground to 150 mesh showed optimal quality and functionality, providing a theoretical basis for its application in functional foods.

## Highlights

• Superfine grinding provides a promising processing strategy for *F. suspensa* fruit.
• Superfine powders showed brighter color, improved hydration, and better flavor.
• Superfine grinding enhanced bioactive compound extractability and bioactivity.
• Grinding *F. suspensa* fruit to 150 mesh resulted in the optimal overall quality.

## Graphical abstract

**Figure:**
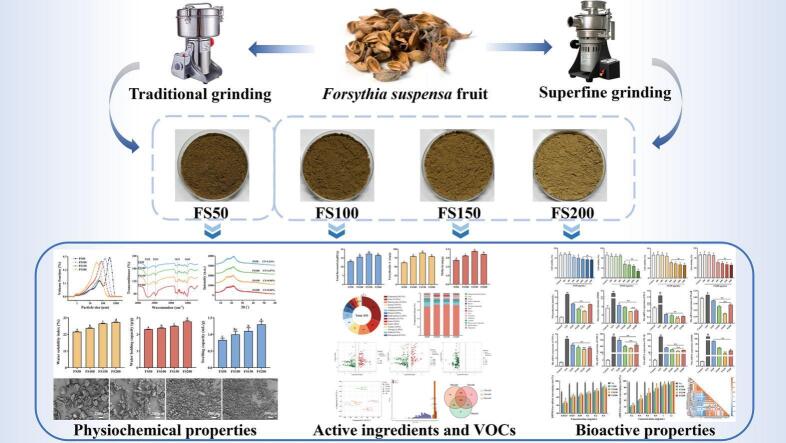

## 1 Introduction

*Forsythia suspensa* (Thunb.) Vahl (*F. suspensa*) is a shrub of the Oleaceae family that has been used in China for nearly a thousand years. Its fruit is rich in bioactive compounds such as phenylethanoid glycosides, flavonoids, terpenoids, and volatile oils, exhibiting notable anti-inflammatory, antibacterial, antioxidant, antiviral, and neuroprotective activities (Li et al., 2024). Owing to these outstanding bioactive properties, *F. suspensa* fruit has been widely used in pharmaceuticals, natural preservatives, and cosmetics (Wang et al., 2019; Wang et al., 2023). In addition, in traditional Chinese folk applications, *F. suspensa* fruit is often incorporated into specialty teas and blended tea bag products to provide both distinctive flavor and health benefits, similar to the use of its leaves as functional teas (Li et al., 2025). The application of *F. suspensa* fruit in functional foods is limited by low extractability of bioactive compounds, bitter taste, and a lack of standardized processing methods. Moreover, research on processing technologies remains limited, highlighting the need to explore advanced techniques to enhance its functional properties.

Superfine grinding is an emerging physical processing technology that has been widely applied to improve food utilization and pharmaceutical production. This technique reduces the particle size of raw materials to the micron, submicron, or even nanometer scale through mechanical shearing, impact, and hydrodynamic forces. For functional foods, particle size is a critical factor influencing powder properties and quality. In plant powders, most bioactive compounds are encapsulated within cells. Reducing particle size increases the surface area and promotes cell wall disruption, thereby enhancing the exposure and extractability of bioactive compounds. Li et al. (2026) found that superfine grinding disrupted the cohesion and structure of the raw material, increasing the surface area, reducing crystallinity, and efficiently breaking cell walls, thereby altering the physical properties and functional characteristics of ginger powder. Al-Riyami et al. (2026) observed that superfine grinding disrupted cellulose, hemicellulose, and other cell wall components, leading to cell wall breakdown and increased exposure of intracellular bioactive compounds and hydrophilic groups, thereby facilitating their efficient extraction. Although superfine grinding may reduce powder flowability and cause thermal degradation of some components, it overcomes processing limitations and offers high efficiency, labor saving, and low cost, showing great potential in functional food development.

Many studies have been conducted on *F. suspensa* fruit; however, limited research has explored the effects of superfine grinding on its quality and functionality. To date, only Weng et al. (2022) have investigated *F. suspensa* leaf, finding that reducing particle size not only altered the physicochemical properties of the powder, but also increased the compound extractability and antioxidant activity. The effects of superfine grinding are closely related to the properties of the material. While most studies indicate that reducing particle size can improve powder performance, Troilo et al. (2022) reported that the content of phenolic compounds in grape pomace is higher in coarser particles. To provide a more systematic and comprehensive evaluation of the effects of superfine grinding on *F. suspensa* fruit, this study prepared four powders of different particle sizes using both traditional and superfine grinding. Based on its distinctive aroma profile and pharmacological activity, this study incorporated volatile compounds (VOCs) analysis and in vitro anti-inflammatory evaluation in addition to conventional assessments of physicochemical properties and antioxidant activity. Furthermore, the correlations among various parameters were clarified, and the optimal grinding degree of *F. suspensa* fruit was determined. This study provides a theoretical basis for the powder processing of *F. suspensa* fruit, thereby enhancing its product value and broadening its application potential.

## 2 Materials and methods

### 2.1 Materials and chemicals

Dried *F. suspensa* fruit was purchased from Hubei Yaosheng Chinese Medicine Technology Co., Ltd., with the production batch number 20240701. The fruit was harvested in July 2024 from Shanxi Province, China, steam-processed, and naturally sun-dried to obtain whole dried *F. suspensa* fruit. Analytical grade chemical reagents, such as sodium nitrite (NaNO_2_), aluminum nitrate (Al(NO₃)₃), sodium hydroxide (NaOH), ethanol (CH_3_CH_2_OH), methanol (CH_3_OH), and potassium persulfate (K_2_S_2_O_8_) were purchased from Sinopharm Chemical Reagents Co., Ltd., China. ABTS (2,2′-azino-bis (3-ethylbenzothiazoline-6-sulfonate) and Vitamin C (VC) were purchased from Shanghai Yuanye Bio-Technology Co., Ltd., China. DPPH (2,2-Diphenyl-1-picrylhydrazyl) was purchased from Shanghai Macklin Biochemical Co., Ltd., China. LPS (Lipopolysaccharide, L2880) was purchased from Sigma-Aldrich Co., Ltd., Germany. HPLC-grade standard compounds, rutin (B25342, purity ≥98%), forsythoside A (24,120,112, purity ≥98%) and phillyrin (25,090,211, purity ≥99%) were purchased from Beifang Weiye Metrology Group Co., Ltd., China, and stored in a refrigerator at 4 °C.

### 2.2 Preparation of powder

The dried *F. suspensa* fruit was traditionally ground using a universal grinder (FW250, Zhengzhou Kefeng Instrument and Equipment Co., Ltd., Zhengzhou, China) and passed through a 50-mesh sieve (355 μm) (GB/T6003.1–2022, Xinxiang Zhongtai Machinery Co., Ltd., Xinxiang, China) to obtain coarse *F. suspensa* fruit powder FS50. The FS50 was further ground in the air-jet micro-pulverizer (WF-18, Wenzhou Dingli Instrument Co., Ltd., Wenzhou, China) with the feeding rate of 10 g/min under room temperature (25 ± 2 °C). To avoid excessive machine load and temperature, the grinding was performed intermittently with 30-s pauses after each 1-min cycle, and the current was controlled to not exceed 15 A. After six cycles, the powder was passed through a series of standard sieves with mesh sizes of 100 (150 μm), 150 (100 μm), and 200 (75 μm) to obtain FS100, FS150 and FS200.

### 2.3 Particle size distribution

Powders were dispersed in deionized water, then a laser particle size analyzer (Mastersizer 3000+ Ultra, Malvern Panalytical, United Kingdom) was used to measure particle size parameters across a size range of 0.01 to 3500 μm. Particle size was described using *D*_10_, *D*_50_, and *D*_90_, which represent the equivalent particle size at cumulative volumes of 10%, 50%, and 90%, respectively. Additionally, the particle size distribution span was calculated as follows.

(1) $$\mathrm{Span}=\left(D90-D10\right)/D50$$

It is generally accepted that the diameter of plant cells ranges from approximately 10 to 20 μm. The cell wall breakage rate (*Φ*) was estimated as follows when *D*_50_ exceeded 10 μm (Zhang et al., 2021).

(2) $$\Phi =1-{\left(1-10/D50\right)}^{3}$$

### 2.4 Specific surface area (SSA) and pore structure

The SSA and pore structure of the powder samples were characterized by nitrogen adsorption–desorption analysis using an automatic surface area and porosity analyzer (Micromeritics ASAP 2460, USA). Measurements were conducted at 77 K. Prior to analysis, approximately 0.8 g of each sample was degassed at 100 °C for 12 h to remove residual gases and moisture.

### 2.5 Color parameters

The color parameters *L*^*⁎*^ (lightness), *a*^*⁎*^ (from green to red), and *b*^*⁎*^ (from blue to yellow) were measured using a color difference meter (LS173, Shenzhen Linshang Technology Co., Ltd., Shenzhen, China) with a full-spectrum LED light source and an 8 mm aperture. Calibration was performed using a standard white plate (*L*^⁎^ = 97.09, *a*^⁎^ = −0.45, *b*^⁎^ = −0.13). The total color difference (Δ*E*) was calculated as follows.

(3) $$\Delta E=\sqrt{{\left(L^{*}-L_{0}\right)}^{2}+{\left(a^{*}-a_{0}\right)}^{2}+{\left(b^{*}-b_{0}\right)}^{2}}$$

Where L_0_, a_0_, and b_0_ were the color parameters of FS50; *L*^⁎^, *a*^⁎^, and *b*^⁎^ were color parameters of other powders.

### 2.6 Scanning electron microscopy (SEM)

Powder samples were mounted on aluminum stubs using conductive carbon tape and then sputter-coated with a 10 nm layer of gold under vacuum. SEM (ZEISS-Sigma 300, Germany) was used to observe the surface morphology of different powders at an acceleration voltage of 15 kV, and the morphology was imaged at magnifications of 50× and 3000 × .

### 2.7 Flow property

#### 2.7.1 Tap density

Tap density (*ρ*_tap_) was measured according to the method of Qadri et al. (2023). A 10 mL volumetric flask was weighed (*m*_1_, g) and then filled with powder samples. The flask was tapped until the powder reached the calibration mark, after which the combined mass of the flask and sample was recorded (*m*_2_, g). Tap density (*ρ*_tap_) was calculated as follows.

(4) $$\rho_{\mathrm{tap}}=\left(m2-m1\right)/10$$

#### 2.7.2 Angle of repose

The angle of repose (*α*) was evaluated using the method described by Zhang et al. (2020). A funnel was fixed vertically above graph paper, and the height (*H*, cm) from the outlet to the paper was recorded. The powder was allowed to flow freely through the funnel to form a cone. When the apex of the cone reached the funnel outlet, the radius (*R*, cm) of the base was measured. The angle of repose (*α*) was calculated as follows.

(5) $$\alpha =\arctan\left(H/R\right)$$

#### 2.7.3 Angle of slide

The angle of slide (*β*) was measured as described by Zhang et al. (2020). A glass plate measuring 7.5 cm (*H*) by 3.5 cm (*W*) was placed horizontally on the tabletop with one end fixed and 1 g of powder was placed on the free end. The free end was lifted gradually until the powder began to slide, and the height (*L*, cm) at this point was recorded. The angle of slide (*β*) was calculated as follows.

(6) $$\beta =\arcsin\left(H/L\right)$$

### 2.8 Hydration property

#### 2.8.1 Water solubility index (WSI)

WSI was conducted according to the method adapted from Weng et al. (2022) with minor modifications. Powders (*m*_1_, g) were mixed with distilled water at a ratio of 1:50 and heated in an 80 °C water bath for 30 min. After centrifuging at 5000 r/min for 10 min, the supernatants were transferred to a pre-weighed evaporating dish (*m*_2_, g) and dried at 105 °C to a constant weight (*m*_3_, g). WSI was calculated as follows.

(7) $$\mathrm{WSI}\left(\%\right)=\left(m3-m2\right)/m1\times 100$$

#### 2.8.2 Water holding capacity (WHC)

WHC was measured as described by Yu et al. (2024). Powders (*m*_1_, g) were placed in centrifuge tubes and weighed (*m*_2_, g). Distilled water was added at a ratio of 1:50 and the mixture was kept at 37 °C for 12 h. After centrifugation at 4000 r/min for 10 min, the supernatant was removed and the precipitate was weighed (*m*_3_, g). WHC was calculated as follows.

(8) $$\mathrm{WHC}\left(g/g\right)=\left(m3-m2\right)/m1$$

#### 2.8.3 Swelling capacity (SC)

SC was assessed using the method described by Weng et al. (2022). 1 g of sample powder (*m*, g) was placed into a graduated test tube and mixed with 10 mL of distilled water. After thorough shaking, the initial volume (*V*_1_, mL) was recorded. The final volume of the precipitate (*V*_*2*_, mL) was recorded after being kept at room temperature (25 ± 2 °C) for 24 h. SC was calculated as follows.

(9) $$\mathrm{SC}\left(\mathrm{mL}/g\right)=\left(V2-V1\right)/m$$

### 2.9 Fourier transform infrared spectroscopy (FTIR) analysis

The powders were vacuum-dried at 60 °C for 24 h to remove moisture, then thoroughly mixed with potassium bromide (KBr) at a 1:100 ratio and compressed into a translucent pellet. After background correction with pure KBr, all FTIR spectra were recorded in the range of 4000 to 400 cm^−1^ at an optical resolution of 4 cm^−1^ using a Nicolet 6700 FTIR spectrometer (Thermo Fisher Scientific, USA).

### 2.10 X-ray diffraction (XRD) analysis

The XRD patterns of the different powders were determined by an X-ray diffractometer (Bruker-D8 ADVANCE, Germany). The radiation source was Cu Kα (λ = 0.15406 nm), with a working current of 40 mA and a voltage of 40 kV. The scanning range was 5–60° with a scan rate of 5°/min. The crystallinity index (CI) of each powder was calculated using Origin2024 software.

### 2.11 Bioactive components content

#### 2.11.1 Total flavonoids content

Rutin standard solution (100 μg/mL) was prepared using 70% ethanol. Standard solutions with volumes 0, 1, 2, 3, 4, 5, and 6 mL were transferred into 25 mL volumetric flasks and the remaining steps were performed according to the NaNO_2_-Al(NO_3_)_3_ method described by Liu et al. (2025). The absorbance was measured at 510 nm using a UV–Vis spectrophotometer (UV-1800PC, Shanghai Meipuda Instrument Co., Ltd.). The regression equation for rutin was y = 0.5028× + 0.0041 (*R*^2^ = 0.999).

The extraction method for the total flavonoids in different powders was adapted from Xiang et al. (2024) with minor modifications. 1 g of the powder was mixed with 20 mL of 70% methanol and ultrasonically treated (250 W, 40 kHz, KQ-400KDE, Kunshan Ultrasonic Instrument Co., Ltd.) for 30 min. After cooling, 70% methanol was added to compensate for weight loss. The content of total flavonoids in the extracts was determined using the aforementioned NaNO_2_-Al(NO_3_)_3_ method and expressed as mg rutin equivalents (RE)/g.

#### 2.11.2 Phillyrin and forsythoside a content

2 g of each powder sample was mixed with 25 mL of methanol and re-weighed. The mixture was extracted by ultrasonication (250 W, 40 kHz) for 30 min. After cooling, methanol was added to compensate for weight loss. The supernatant was diluted 30-fold and filtered through a 0.22 μm PES membrane to obtain the test solution. The standards of phillyrin and forsythoside A were dissolved in methanol to prepare stock solutions with concentrations of 56.5 μg/mL and 300.9 μg/mL. The mixed solution was serially diluted to prepare a series of standard solutions with phillyrin concentrations of 28.25, 14.13, 7.06, 3.53, and 1.77 μg/mL, and forsythoside A concentrations of 150.45, 75.23, 37.61, 18.81, and 9.40 μg/mL.

High-performance liquid chromatography (HPLC) analysis was performed using a C_18_ column (4.6 mm × 250 mm, 5 μm, Agilent, 770450–902) equipped with C_18_ guard columns (4.6 mm × 10 mm, 5 μm) on an Agilent 1200 HPLC system (Agilent Technologies, Santa Clara, CA, USA). The mobile phase, consisting of acetonitrile (A) and 0.1% formic acid aqueous solution (B), was filtered through a 0.40 μm nylon membrane to remove impurities. The elution gradient was as follows: 0–15 min, 18% ∼ 25% A; 15–25 min, 25% ∼ 40% A. The flow rate was 1.0 mL/min, the column temperature was 30 °C, and the injection volume was 10 μL. Based on gradient concentration standard solutions, calibration curves were constructed and showed excellent linearity. For forsythoside A, the regression equation was y = 6.4368× - 11.752 (*R*^2^ = 0.999), while for phillyrin, it was y = 3.3553× + 1.928 (*R*^2^ = 0.999). The contents of phillyrin and forsythoside A in the extracts were determined using the corresponding regression equations.

### 2.12 VOCs analysis

VOCs were analyzed using headspace solid-phase microextraction coupled with gas chromatography–mass spectrometry (HS-SPME-GC–MS), following a previously reported method with minor modifications (Xie et al., 2025). Briefly, 0.2 g of powder and 0.2 g of NaCl were placed into a 20 mL headspace vial and sealed with a PTFE–silicone septum. After equilibration at 60 °C for 5 min, a 120 μm DVB/CWR/PDMS SPME fiber (Agilent, USA) was exposed to the headspace at 60 °C for 15 min.

After extraction, VOCs were thermally desorbed in the GC apparatus (Model 8890; Agilent) at 250 °C for 5 min in splitless mode. GC–MS analysis was conducted using an Agilent 8890 GC coupled with a 7000D MS detector equipped with a DB-5MS capillary column (30 m × 0.25 mm × 0.25 μm). Helium served as the carrier gas at 1.2 mL/min. The oven program was set as follows: 40 °C for 3.5 min; ramped at 10 °C/min to 100 °C, 7 °C/min to 180 °C, and 25 °C/min to 280 °C, holding for 5 min. The MS operated in EI mode (70 eV) under SIM acquisition with ion source, quadrupole, and transfer-line temperatures of 230, 150, and 280 °C, respectively.

VOCs identification and quantification followed the Metware database (Wuhan, China). One quantitative ion and 2–3 qualitative ions were selected per compound. Peaks matching reference retention times and ion fragments were considered positive identifications. Quantitative ions were integrated using MassHunter software.

### 2.13 Anti-inflammatory activity

According to the method described by Mu et al. (2024), 10 g powder was mixed with 200 mL of 75% ethanol, then heated and refluxed twice, each for 30 min. The filtrate was then concentrated using a rotary evaporator (RE-52 A, Shanghai Yarong Biochemical Instrument Factory, China) and freeze-dried to obtain the crude extract.

RAW264.7 mouse macrophages (1 × 10^5^ cells/mL, 100 μL/well) were inoculated into a 96-well plate, and maintained in an incubator (HF151/212, Heal Force, Chenguang Technology Co., Ltd., Baoji, China) with 5% CO_2_ at 37 °C for 24 h. The medium was then replaced with *F. suspensa* powder extracts (200–1400 μg/mL) and incubated for another 24 h. Cell viability was measured using the MTT method to determine the optimal concentration.

After inoculating RAW264.7 cells onto plates using the same method described above and incubating for 24 h, the experimental groups were treated with 100 μL of four *F. suspensa* powder extracts (600 μg/L). After 3 h, LPS (100 ng/mL) was added for a 24 h stimulation. Normal RAW264.7 cells were used as the control group, and cells treated with LPS alone were used as the model group. NO and ROS were measured according to the instructions of the assay kit. Total RNA in cells was extracted using TRNzol Universal and qRT-PCR was performed to determine the mRNA expression levels of the target genes. Primer sequences are listed in Table S1.

### 2.14 Antioxidant activities

#### 2.14.1 DPPH free radical scavenging capacity

The different powder extracts were dissolved in ethanol and diluted to prepare solutions at concentrations of 0.0125, 0.025, 0.05, 0.10, 0.20 and 0.40 mg/mL. DPPH radical scavenging assay was performed according to Xiao et al. (2024). In brief, equal volumes of sample solution and DPPH solution (0.1 mmol/L) were mixed and incubated in the dark for 30 min. Then, the absorbance (*A*_2_) was measured at 517 nm. The control group (*A*_1_) consisted of ethanol and DPPH solution, and the blank group (*A*_0_) included the sample solution and ethanol. VC was used as the positive control. The scavenging rate was calculated as follows.

(10) $$\mathrm{DPPH radical scavenging rate}\;\left(\%\right)=\left(1-\left(A2-A1\right)/A0\right)\times 100$$

#### 2.14.2 ABTS free radical scavenging capacity

Powder extracts were dissolved in deionized water and diluted to 0.2, 0.4, 0.8, 1.0, and 1.2 mg/mL. ABTS radical scavenging assay was evaluated using the method of Wang, Liu, et al. (2025). In brief, equal volumes of potassium persulfate solution (2.45 mmol/L) and ABTS (7 mmol/L) solution were mixed and incubated in the dark for 12 h, then diluted to an absorbance of 0.7 ± 0.02 at 734 nm. Sample solution (0.1 mL) was mixed with ABTS solution (3.9 mL) and placed in the dark for 6 min. Absorbance (*A*_2_) was then measured at 734 nm. The control group (*A*_1_) consisted of the sample solution and deionized water, while the blank group (*A*_0_) consisted of deionized water with ABTS solution. VC was used as the positive control. The scavenging rate was calculated as follows.

(11) $$\mathrm{ABTS free radical scavenging rate}\;\left(\%\right)=\left(1-\left(A2-A1\right)/A0\right)\times 100$$

### 2.15 Statistical analysis

All data were analyzed using SPSS (version 19.0, SPSS Inc., Chicago, IL, USA) and subjected to one-way analysis of variance (ANOVA) by Tukey's post hoc test with a 95% confidence level. The results were presented using mean ± standard deviation (SD). Except for the cell experiments (*n* = 6), all other experiments were performed in triplicate (*n* = 3), and *P* < 0.05 was considered statistically significant. Correlation coefficients (*r*) between the variables were calculated by Pearson's correlation method. The experimental data were plotted using Origin software (version 2024, OriginLab Corporation, Northampton, MA, USA) and GraphPad Prism software (version 10.4.0, GraphPad Software, Inc., San Diego, CA, USA).

## 3 Results and discussion

### 3.1 Particle size distribution

**Fig. 1:**
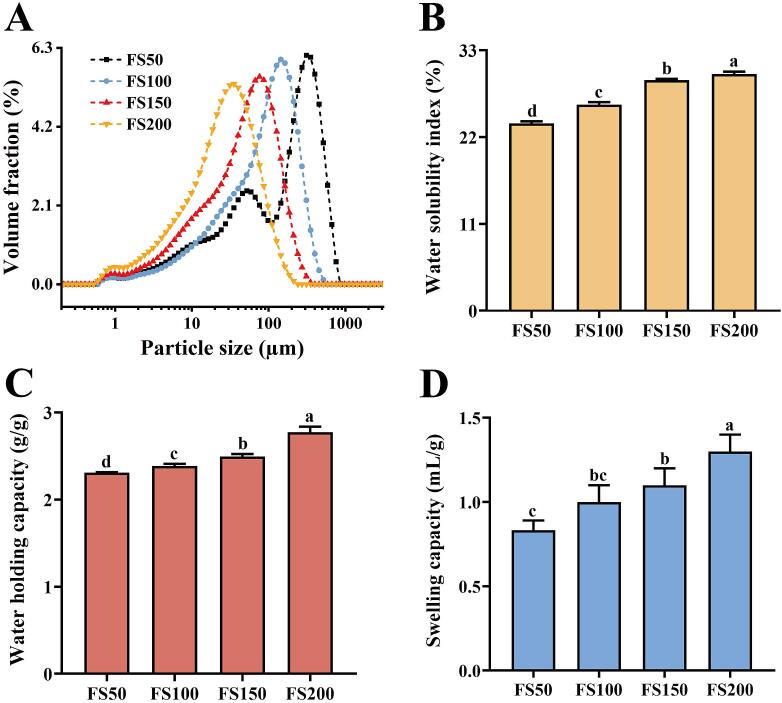
Particle size distribution (A) and hydration property of the *F. suspensa* fruit powders. (B) Water solubility index; (C) water holding capacity; (D) swelling capacity. Values with different superscript letters in the same graph were significantly different (*P* < 0.05).

The particle size parameters of the powders were summarized in Table 1. The *D*_50_ decreased from 185.33 μm (FS50) to 99.93 μm (FS100), 53.20 μm (FS150), and 27.93 μm (FS200), which confirmed that superfine grinding effectively reduced particle size. The span value was highest in FS200 (2.80), indicating a broader particle size distribution and reduced uniformity, consistent with the distribution curves in Fig. 1A and previous findings (Weng et al., 2022). However, FS100 (2.46) showed a lower span value than FS50 (2.62). This was likely because FS100 was prepared by air-jet milling of FS50, which preferentially broke large particles, and the 100-mesh sieve more effectively removed coarse particles, resulting in a more concentrated distribution, consistent with Duguma et al. (2023). Additionally, the *Φ* values gradually increased from FS50 (15.33%) to FS200 (73.54%), representing a 79.15% increase, suggesting that superfine grinding enhanced the cell wall breakage rate.

**Table 1:** Particle size parameters and pore structure parameters of the *F. suspensa* fruit powders.

Samples	*D*_10_ (μm)	*D*_50_ (μm)	*D*_90_ (μm)	*Span*	*Φ* (%)	SSA (m^2^/g)	TPV (cm^3^/g)	APD (nm)
FS50	13.03 ± 0.06^a^	185.33 ± 3.2^a^	497.00 ± 10.25^a^	2.62 ± 0.03^b^	15.33 ± 0.25^d^	0.0672	0.007308	6.8014
FS100	11.37 ± 0.21^b^	99.93 ± 0.12^b^	257.00 ± 2.65^b^	2.46 ± 0.03^c^	27.12 ± 0.03^c^	0.1130	0.009797	6.2127
FS150	7.26 ± 0.01^c^	53.20 ± 0.10^c^	147.67 ± 0.58^c^	2.64 ± 0.01^b^	46.46 ± 0.07^b^	2.0663	0.008851	6.4125
FS200	4.70 ± 0.02^d^	27.93 ± 0.15^d^	83.00 ± 0.61^d^	2.80 ± 0.01^a^	73.54 ± 0.24^a^	4.2269	0.007587	6.6719

The results were expressed as mean of three determinations ± standard deviation. Different lowercase superscripts in the same column were significantly different (*P* < 0.05).

### 3.2 SSA and pore structure

As shown in Table 1, the SSA gradually increased from 0.0672 m^2^/g (FS50) to 4.2269 m^2^/g (FS200), representing an approximately 63-fold enhancement. This result indicated that superfine grinding effectively increased the exposed surface area of the powders, which had a positive influence on the digestion rate and bioavailability of food powders (Wang, Zhao, et al., 2025). The superfine powders showed higher total pore volume (TPV) values than FS50, indicating that superfine grinding promoted pore development and improved pore accessibility. FS100 exhibited the highest TPV value (0.009797 cm^3^/g), whereas FS150 (0.008851 cm^3^/g) and FS200 (0.007308 cm^3^/g) showed slight decreases, which may be associated with partial pore collapse and particle agglomeration caused by excessive grinding (Kapadnis et al., 2025). The average pore diameter (APD) of all powders ranged from 6.21 to 6.80 nm, indicating a typical mesoporous structure (2–50 nm). The nitrogen adsorption–desorption isotherms (Fig. S1A—D) exhibited type IV isotherms accompanied by a weak H3 hysteresis loop, further confirming the mesoporous nature of the powders and indicating that the pore structure was mainly composed of slit-shaped pores and interparticle voids. Compared with FS50, the superfine powders exhibited reduced APD values, suggesting that superfine grinding promoted the fragmentation and refinement of larger pores. However, the APD values of FS150 and FS200 increased slightly with further grinding, which may be attributed to particle aggregation and the formation of interparticle voids (Wu et al., 2026).

### 3.3 Color difference

**Fig. 2:**
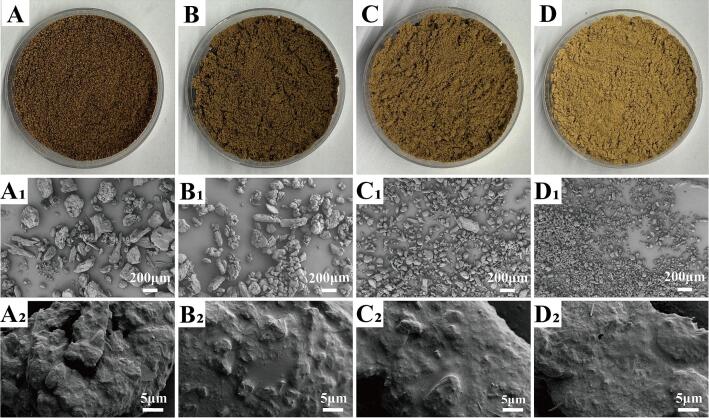
Actual and SEM images of the *F. suspensa* fruit powders. Actual images: (A) FS50; (B) FS100; (C) FS150; (D) FS200; SEM images: (A_1_) FS50, 50×; (B_1_) FS100, 50×; (C_1_) FS150, 50×; (D_1_) FS200, 50×; (A_2_) FS50, 3000×; (B_2_) FS100, 3000×; (C_2_) FS150, 3000×; (D_2_) FS200, 3000 × .

As shown in Table 2, the *L*^*⁎*^ and *b*^*⁎*^ values increased progressively from FS50 to FS200, suggesting that finer particle size enhanced powder brightness and yellowness. This trend may be attributed to increased light reflection caused by the larger surface area of finer particles (Vela et al., 2024) and the release of yellow pigments and phenolic aggregates following cell disruption (Hong et al., 2021). The *a*^*⁎*^ value generally increased with decreasing particle size; however, the *a*^*⁎*^ value of FS200 was comparable to FS50, which may be attributed to chlorophyll release after cell rupture (Liu et al., 2024). Jiang et al. (2020) also reported similar results in their study on onion skin powder, where smaller particles exhibited higher *L*^*⁎*^ and *b*^*⁎*^ values and lower *a*^*⁎*^ values. The Δ*E* increased significantly with the decrease in particle size, which was consistent with the findings of (Zhang et al., 2021). As shown in Fig. 2A–D, the superfine powders exhibited more vivid colors and exhibited smoother surfaces after superfine grinding, which may improve the acceptability of *F. suspensa* fruit and promote its application in the food industry.

**Table 2:** Color parameters, tap density, angle of repose and slide of the *F. suspensa* fruit powders.

Samples	*L*^*⁎*^	*a*^*⁎*^	*b*^*⁎*^	Δ*E*	*ρ*_tap_ (g/mL)	*α* (^°^)	*β* (^°^)
FS50	50.48 ± 0.05^d^	5.10 ± 0.06^c^	14.43 ± 0.07^d^	0.00^d^	0.50 ± 0.01^c^	44.63 ± 1.89^c^	21.71 ± 4.04^d^
FS100	52.81 ± 0.06^c^	5.50 ± 0.03^b^	15.78 ± 0.07^c^	2.72 ± 0.08^c^	0.51 ± 0.01^b^	50.55 ± 2.37^b^	25.47 ± 3.55^c^
FS150	55.61 ± 0.05^b^	5.81 ± 0.03^a^	18.90 ± 0.04^b^	6.84 ± 0.02^b^	0.52 ± 0.01^a^	50.09 ± 1.85^b^	33.54 ± 2.14^b^
FS200	62.58 ± 0.03^a^	5.05 ± 0.03^c^	21.79 ± 0.04^a^	14.16 ± 0.04^a^	0.53 ± 0.01^a^	54.88 ± 1.51^a^	39.24 ± 2.68^a^

The results were expressed as mean of three determinations ± standard deviation. Different lowercase superscripts in the same column were significantly different (*P* < 0.05).

### 3.4 Powder morphology characterization

Powder morphology and SEM images were shown in Fig. 2. Compared with the coarse powder FS50, the superfine powders exhibited increased agglomeration, which may be related to the larger SSA and electrostatic effects (Gan et al., 2023). The SEM images revealed that superfine grinding markedly reduced particle size and altered particle morphology. Specifically, the superfine powders exhibited more regular particle shapes, a higher proportion of spherical structures, and smoother surfaces. This finding aligned with the particle size distribution results shown in Fig. 1A. Overall, superfine grinding markedly altered the morphology and microstructure of the powder, which may affect its physicochemical and functional properties (Jiang et al., 2020).

### 3.5 Flow property

Flowability is an important property of powders that influences powder quality and is evaluated by tap density (*ρ*_tap_), angle of repose (*α*), and angle of slide (*β*). As shown in Table 2, all three parameters significantly increased, indicating that the flowability of the powders deteriorated. These results may be attributed to the reduced interparticle gaps after superfine grinding, which enhanced electrostatic attraction and van der Waals forces between particles, thereby hindering relative particle movement (Du et al., 2022). The reduced flowability may play a positive role in the processes of dosing, sieving, and blending of powdered foods such as instant beverages and soup mixes (Jiang et al., 2020).

### 3.6 Hydration property

WSI reflects the solubility of components in the powder. As shown in Fig. 1B, WSI increased from 23.74% to 29.99% with decreasing particle size, which may be related to the differences in the soluble dietary fiber content (Li et al., 2026). WHC can assess the ability of powder to retain moisture when subjected to external forces, reflecting the strength of the interaction between the material and water. As shown in Fig. 1C, WHC increased from 2.31 g/g (FS50) to 2.77 g/g (FS200). SC, which can reflect the water absorption capacity, was significantly higher in FS100, FS150, and FS200 than in FS50 (*P* < 0.05) (Fig. 1D). As noted by Zhang et al. (2020), the larger SSA of superfine powders exposed more hydrophilic groups and enhanced the interaction between the powder and water. Improved hydration properties of the powders can prevent product shrinkage and regulate system viscosity, facilitating the broader application of *F. suspensa* fruit in solid beverages, meal replacements, and gel-based products.

### 3.7 FTIR

**Fig. 3:**
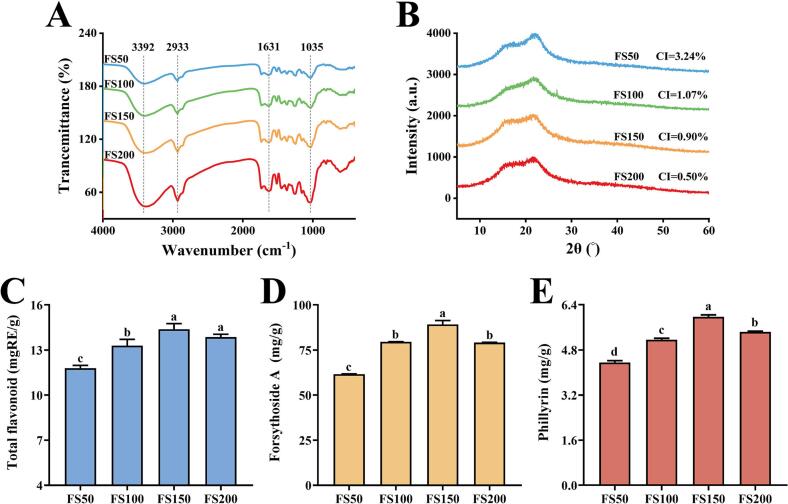
FTIR (A), XRD (B) and bioactive ingredient content of the *F. suspensa* fruit powders. (C) Total flavonoids; (D)forsythoside A; (E) phillyrin. Values with different superscript letters in the same graph were significantly different (*P* < 0.05).

FTIR was used to analyze the functional group characteristics of the compounds, and the spectral results were shown in Fig. 3A. All four powders exhibited significant characteristic bands at 3392 cm^−1^, 2933 cm^−1^, 1631 cm^−1^, and 1035 cm^−1^. The absorption band at 3392 cm^−1^ was attributed to the complex stretching vibrations of intermolecular and intramolecular O—H groups in cellulose and hemicellulose, suggesting the presence of cellulose and hemicellulose in the powder (Li et al., 2026). The peak at 2933 cm^−1^ was attributed to asymmetric C—H stretching vibrations in sugar groups (Yu et al., 2024). The absorption at 1631 cm^−1^ corresponded to the C=O vibration of the amide group (Sharma et al., 2025), while the band at 1035 cm^−1^ was assigned to the C—O stretching or bending vibrations, which is associated with pyranose rings and represented characteristic absorptions of polysaccharides such as lignin and cellulose (Kang et al., 2024). The nearly identical spectra of all powders indicated that no new chemical groups were generated during grinding. However, with decreasing particle size, the peak intensity gradually increased. This may be attributed to the disruption of hydrogen bonds between cellulose and hemicellulose during superfine grinding, leading to the formation of soluble sugars and amorphous cellulose (Liang et al., 2022), which was further supported by the XRD results (Section 3.7). Additionally, the characteristic peak intensities of hydrophilic groups such as O—H and C—O gradually increased, indicating enhanced exposure of these groups. Exposed O—H bonds formed stronger hydrogen bonds with water molecules to enhance water adsorption and retention, while the exposed polysaccharide-based hydrophilic structures corresponding to C—O bonds promoted water-soluble component dissolution and particle swelling, collectively improving the WSI, WHC and SC. Furthermore, the exposure and transformation of hydrophilic groups not only enhanced the hydration properties of the powders but also promoted the dissolution of bioactive compounds (Section 3.8).

### 3.8 XRD

XRD patterns reflect the crystalline properties of different powders. As shown in Fig. 3B, all powders exhibited similar diffraction patterns, indicating that superfine grinding did not markedly alter the main composition of the powders. No sharp diffraction peaks were observed, suggesting a low overall crystallinity of the *F. suspensa* fruit powders. All four powders exhibited a broad amorphous peak at diffraction angles (2θ) of approximately 10–26°, indicating that the samples were predominantly amorphous, which was closely related to the presence of non-crystalline cellulose, hemicellulose, and lignin in the powders (Cao et al., 2024). Compared with FS50, three superfine powders exhibited slightly reduced amorphous peak intensities and lower CI values, a trend also observed in studies on milled foxtail millet flour (Y. T. Wang et al., 2025). This could be attributed to the disruption of hydrogen bonds in the crystalline regions of cellulose and hemicellulose during superfine grinding, leading to their conversion into amorphous structures and soluble sugars (Guo et al., 2025). These structural changes may further influence the hydration properties of the powders and the extractability of bioactive compounds.

### 3.9 Bioactive ingredient content

Forsythoside A, phillyrin, and flavonoids such as rutin and quercetin are key bioactive constituents of *F. suspensa* and exhibit anti-inflammatory and antioxidant activities (Zhou et al., 2024). Thus, quantifying these components is essential for evaluating the effects of superfine grinding on powder quality. As shown in Fig. 3C–E, the contents of total flavonoids, forsythoside A, and phillyrin exhibited similar trends: they increased as particle size decreased, peaked in FS150, and then declined in FS200. Compared with FS50, the total flavonoid content in FS150 increased by 20.98%, forsythoside A by 24.88%, and phillyrin by 28.53%. Compared with FS150, the contents of all three components in FS200 decreased, suggesting that excessive superfine grinding may negatively affect the extractability of bioactive components. A similar pattern was reported for polysaccharide extraction from goji leaves (Quan et al., 2023).

### 3.10 VOCs analysis

#### 3.10.1 Classification analysis of VOCs

**Fig. 4:**
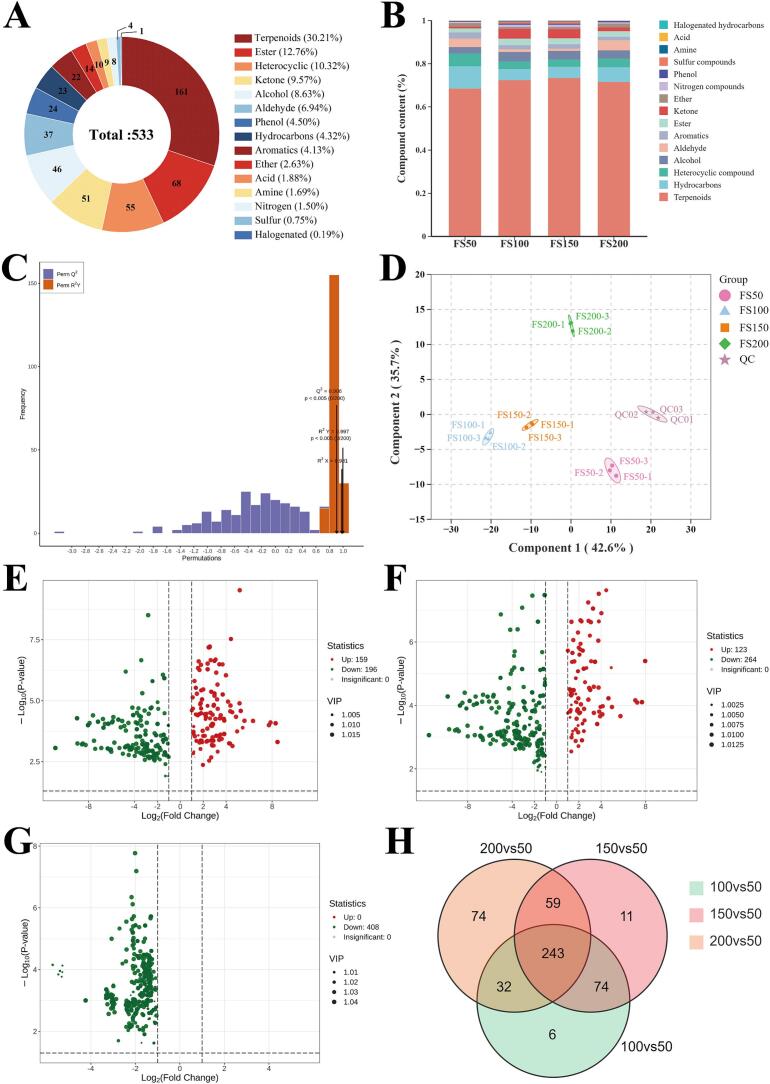
VOCs analysis of the *F. suspensa* fruit powders. (A) VOCs classified in pie charts; (B) the relative contents percentage of VOCs; (C) the permutation test chart for OPLS–DA model; (D) scores of the OPLS–DA model; (E) volcano plots of differential VOCs in FS100 vs FS50; (F) volcano plots of differential VOCs in FS150 vs FS50; (G) volcano plots of differential VOCs in FS200 vs FS50; (H) venn diagram of differential VOCs.

As shown in Fig. 4A, a total of 533 VOCs were identified in the four powders using HS-SPME-GC–MS and classified into 15 categories. Among them, terpenoids (30.21%), esters (12.76%), and heterocyclic compounds (10.32%) were the most diverse, together accounting for over 50% of the compounds. Fig. 4B showed the relative contents of VOCs in different powders. Terpenoids were the most abundant, followed by hydrocarbons, heterocyclic compounds, and alcohols. Notably, from FS50 to FS150, the proportion of terpenoids increased from 68.5% to 72.4% and 73.4%, before slightly decreasing in FS200 (71.5%). Compared with FS50, terpenoids increased by 5.7% and 7.1% in FS100 and FS150, respectively.

#### 3.10.2 Differential VOCs analysis

OPLS-DA is a supervised discriminant analysis method that maximizes intergroup differences and distinguishes variations in VOCs among different powders. As shown in Fig. 4C, the model exhibited high predictive performance, with R^2^X = 0.981, R^2^Y = 0.997, and Q^2^ = 0.906 (all close to 1, *P* < 0.05), indicating high model reliability. Clear clustering within groups and distinct separation among groups were observed in the score plot (Fig. 4D), indicating substantial VOCs differences among powders. Based on the OPLS-DA model, differential VOCs were identified using the criteria VIP > 1, log_2_FC > 1, and *P* < 0.05, and volcano plots were generated (Fig. 4E–G). Compared with FS50, each superfine powder exhibited more downregulated than upregulated VOCs, suggesting some VOCs were lost during superfine grinding. FS100 and FS150 showed upregulated VOCs, whereas no upregulated VOCs were detected in FS200. This may be attributed to the reduction in particle size and the increase in surface area, which enhanced the content of certain VOCs. However, excessive grinding led to the loss and degradation of VOCs. Among the upregulated VOCs, the contents of terpenoids and hydrocarbons increased significantly, including 2,6-octadiene, 2,6-dimethyl-, cis-2,6-dimethyl-2,6-octadiene, 2,6-dimethyl-2trans-6-octadiene, and terpinen-4-ol, which were characterized by fruity, floral, and herbal aromas.

A Venn diagram was produced for the three sets of differential VOCs (Fig. 4H), and 243 VOCs were available to distinguish different powders. These VOCs were mainly classified into terpenoids, aldehydes, ketones, esters, and alcohols. Terpenoids were the most abundant group, accounting for approximately 40%, and played a central role in the aroma differences among powders. Representative terpenoids such as limonene, terpinen-4-ol, β-myrcene, and α-pinene contributed fruity, floral, and citrus-like notes. Aldehydes including hexanal, nonanal, and benzaldehyde provided green and fatty aromas, while ketones and esters such as 3-octanone, methyl salicylate, and ethyl acetate imparted milky and sweet characteristics. In addition, small amounts of alcohols such as linalool and geraniol added floral and fresh nuances. These compositional differences accounted for the distinct aroma profiles of the powders.

#### 3.10.3 Key aroma compounds analysis

The relative odor activity value (rOAV) was used to evaluate the contribution of individual VOCs to the overall aroma and to identify key aroma-active compounds. In general, compounds with rOAV ≥1 were considered to contribute directly to the sample's aroma. Based on this criterion, a total of 241 VOCs were identified as contributing to the aroma of the powders. Moreover, compounds with rOAV ≥10,000 were considered to have an outstanding impact on the overall aroma profile (Xie et al., 2025). Among the four powders, 32 VOCs in FS50, 31 in FS100, 30 in FS150, and 19 in FS200 exhibited rOAV ≥10,000. By taking the intersection of these four groups, 19 common key aroma compounds were identified (Table S2). These VOCs played prominent roles in the overall aroma profile of *F. suspensa* fruit powder, being associated with fresh fruity, floral, roasted, and spicy aroma.

### 3.11 Anti-inflammatory activity

**Fig. 5:**
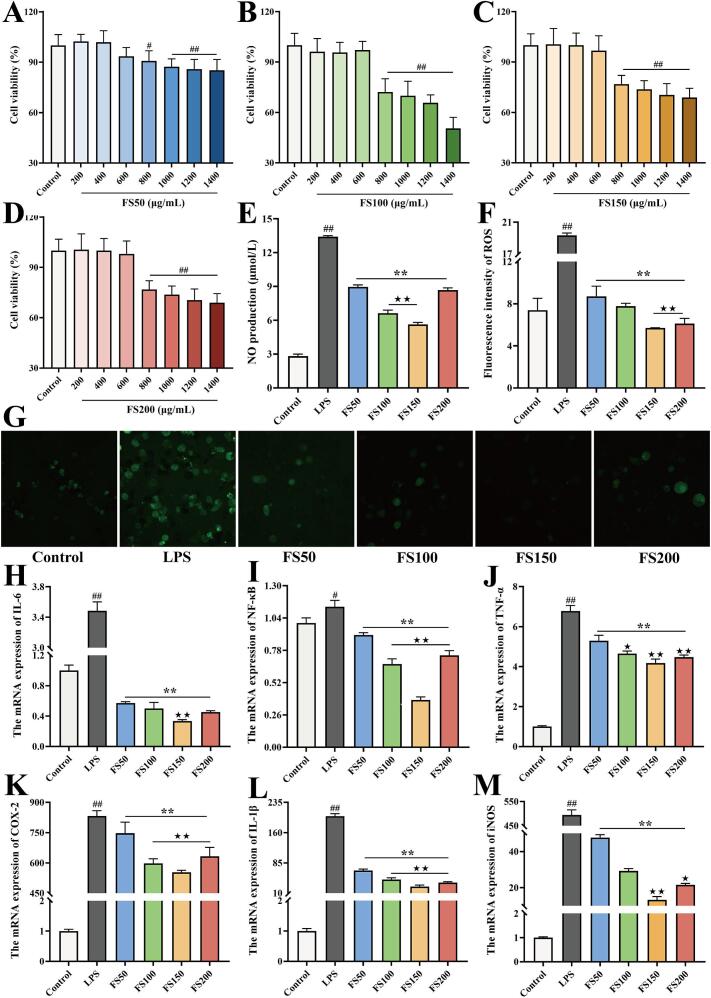
Anti-inflammatory activity of the *F. suspensa* fruit powders. (A-D) Cell viability; (E) NO levels; (F-G) ROS levels; (H-M) the mRNA expression of inflammatory cytokine. ^#^*P* < 0.05, ^##^*P* < 0.01 vs control group; ^⁎^*P* < 0.05, ^⁎⁎^*P* < 0.01 vs LPS group; ^★^*P* < 0.05, ^★★^*P* < 0.01 vs FS50 group.

As shown in Fig. 5A–D, all four powder extracts significantly reduced cell viability at 800 μg/mL and above, indicating that this concentration represented the cytotoxic threshold. Therefore, to minimize potential cytotoxic effects while maximizing the anti-inflammatory activity of the extracts, 600 μg/mL was selected as the treatment concentration for subsequent experiments. NO and ROS are important inflammatory mediators commonly used to assess the degree of inflammation (Cha et al., 2025). During inflammation, their elevation triggers the production and expression of inflammatory cytokines including TNF-α, IL-1β, and IL-6 (Kang et al., 2023). As shown in Fig. 5E and F, LPS stimulation significantly increased NO and ROS levels compared with the control group (*P* < 0.05), whereas treatment with *F. suspensa* powder extracts markedly reduced both indicators compared with the LPS group (*P* < 0.05), with FS150 exhibiting the strongest inhibitory effect. The fluorescence imaging of ROS in Fig. 5G showed consistent results. The mRNA expression levels of IL-6, NF-κB, TNF-α, COX-2, IL-1β, and iNOS are shown in Fig. 5H–M. Treatment with the powder extracts significantly suppressed the expression of these inflammatory cytokines compared with the LPS group (*P* < 0.05), with FS150 exhibiting the strongest effect.

### 3.12 Antioxidant activities

#### 3.12.1 DPPH free radical scavenging capacity

**Fig. 6:**
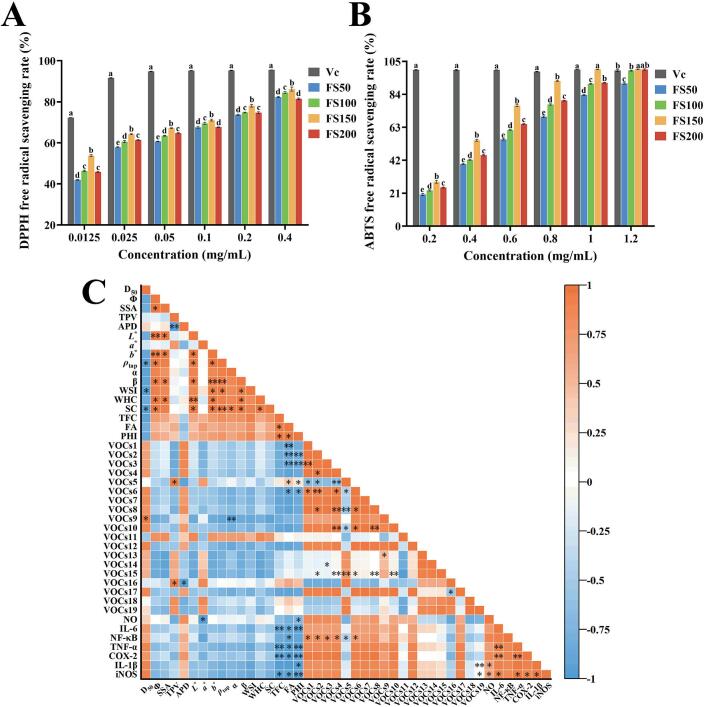
DPPH (A) and ABTS (B) free radical scavenging activity, and correlation analysis among the measured indicators (C). Values with different superscript letters in the same graph were significantly different (*P* < 0.05). * indicates significant correlation (*P* < 0.05), ** indicates highly significant correlation (*P* < 0.01). TFC: total flavonoids content; FA: forsythoside A; PHI: phillyrin.

The antioxidant capacity of the samples can be assessed by its ability to scavenge DPPH free radicals (Yang et al., 2024). As shown in Fig. 6A, the DPPH free radical scavenging activity of all four powders increased with increasing concentration. FS100, FS150, and FS200 exhibited stronger scavenging activity than FS50 across all tested concentrations. Notably, FS150 showed the highest antioxidant capacity, whereas FS100 and FS200 were slightly lower.

#### 3.12.2 ABTS free radical scavenging capacity

A comprehensive evaluation of the antioxidant activity of samples typically requires two or more analytical methods (Bai et al., 2025). Therefore, the ABTS radical scavenging activity was also assessed. As shown in Fig. 6B, all superfine powders exhibited nearly 100% scavenging activity at 1.2 mg/mL. At concentrations below 1.2 mg/mL, FS150 showed the strongest ABTS radical scavenging activity, and FS50 exhibited the weakest activity.

### 3.13 Correlation analysis

The correlations among different indicators were shown in Fig. 6C. *D*_50_ was significantly negatively correlated with *ρ*_tap_, WSI, and SC (*P* < 0.05). In contrast, SSA and *Φ* showed significant positive correlations with multiple physicochemical properties, including *L*^⁎^, *α*, *β*, *b*^⁎^, *ρ*_tap_, WHC, and SC (*P* < 0.05, *P* < 0.01). These results indicate that reduced particle size, increased SSA, and enhanced cell disruption influence powder color, flowability, and hydration properties. Color parameters (*L*^⁎^, *a*^⁎^), flowability parameters (*ρ*_tap_, *α*, *β*), and hydration-related parameters (WSI, WHC, SC) were also mostly positively correlated (*P* < 0.05, *P* < 0.01), further supporting the interdependence of physicochemical properties.

Active components (TFC, FA, PHI) were generally negatively correlated with antioxidant indicators (DPPH, ABTS) and inflammatory markers (NO, NF-κB, IL-6, TNF-α, COX-2, IL-1β, iNOS), suggesting that the accumulation of bioactive compounds underlies the enhanced anti-inflammatory and antioxidant activities. However, some VOCs showed significant positive correlations with anti-inflammatory and antioxidant indicators (*P* < 0.05), indicating that these aroma-contributing compounds may play a limited role in the bioactivity. Notably, antioxidant indicators (DPPH, ABTS) were positively correlated with inflammatory factors (NF-κB, IL-6, TNF-α, IL-1β, iNOS; *P* < 0.05), demonstrating a consistent trend between antioxidant and anti-inflammatory capacities. TNF-α, IL-1β, and iNOS were also significantly correlated, suggesting potential synergistic roles in the anti-inflammatory response.

## 4 Conclusions

In this study, four *F. suspensa* fruit powders with different particle sizes were prepared by traditional and superfine grinding. The results demonstrated that superfine grinding not only improved the color parameters (lightness, yellowness, and greenness) and microstructure of the powders, but also enhanced their physicochemical properties through multidimensional structural regulation. Specifically, the strong mechanical forces generated during superfine grinding markedly reduced particle size, resulting in an approximately 63-fold increase in SSA, a 79.15% increase in *Φ*, and a 34.06% increase in TPV. These changes disrupted the cellular barrier and promoted pore opening, thereby providing mass transfer pathways for solvent penetration. In addition, the grinding process weakened the intermolecular hydrogen bonding interactions between cellulose and hemicellulose, leading to greater exposure of hydrophilic groups, which enhanced the interaction between the powders and water molecules. Meanwhile, the decreased CI indicated the partial transformation of crystalline cellulose into amorphous structures and soluble sugars, which reduced the molecular ordering and further weakened van der Waals forces and hydrogen-bond constraints between molecules. Collectively, these structural changes synergistically improved the hydration properties and solvent accessibility of the powders, thereby promoting the extraction efficiency of key bioactive compounds and enhancing their biological activities. With decreasing particle size, the extraction yields of total flavonoids, forsythoside A, and phillyrin first increased and then decreased, reaching maxima in FS150, which were 20.98%, 24.88%, and 28.53% higher than FS50, respectively. HS-SPME-GC–MS analysis identified 533 VOCs, with terpenoids increasing by 5.7% and 7.1% in FS100 and FS150, respectively. Among the differential VOCs upregulated in FS100 and FS150, terpenoids and hydrocarbons represented the most diverse classes, associated with the powders' distinctive fruity, floral, and herbal aromas. Furthermore, superfine grinding substantially improved the anti-inflammatory and antioxidant activities of the powders, with FS150 exhibiting the strongest capacity. Correlation analysis further revealed that bioactive components were closely associated with these activities. Overall, *F. suspensa* fruit superfine ground to 150 mesh produced powders with optimal physicochemical properties, improved aroma characteristics, and maximized biological potential. This study provides scientific support for its application as a natural flavoring agent or functional ingredient in food systems, including solid beverages and meal replacement products. However, further studies are still needed to investigate in vivo bioactivity, optimize the grinding process, and evaluate sensory properties and applications in functional foods, thereby facilitating its industrial development and large-scale utilization.

## CRediT authorship contribution statement

**Yu Li:** Writing – original draft, Methodology, Formal analysis, Data curation, Conceptualization. **Tianjian Guo:** Methodology, Investigation, Formal analysis, Data curation, Conceptualization. **Ruixi Gao:** Supervision, Investigation, Conceptualization. **Yupeng Liu:** Project administration, Funding acquisition, Conceptualization. **Jun Li:** Supervision, Investigation, Funding acquisition, Conceptualization. **Xiao Chen:** Supervision, Project administration, Funding acquisition. **Ghulam Murtaza:** Writing – review & editing, Supervision, Methodology, Investigation, Conceptualization. **Han Cheng:** Validation, Supervision, Funding acquisition. **Xianju Huang:** Writing – review & editing, Supervision, Project administration, Methodology, Funding acquisition, Conceptualization.

## Declaration of competing interest

The authors declare that they have no known competing financial interests or personal relationships that could have appeared to influence the work reported in this paper.

## Data Availability

Data will be made available on request.
