# Supplementary material for: Superfine grinding on the physicochemical properties, volatile compounds, and bioactive properties of Forsythia suspensa (Thunb.) Vahl fruit powder
Source: Food Chem X. 2026 May 22;36:104018. doi: 10.1016/j.fochx.2026.104018 (PMC13223707; doi:10.1016/j.fochx.2026.104018)
Supplement: Supplementary file 1 — Supplementary material [file mmc1.docx]

**Table S1 Primer sequences used for RT-qPCR.**

| **Gene name** | **Primer sequence5'-3'** |
| --- | --- |
| GAPDH | F：AGGTCGGTGTGAACGGATTTG  R：GGGGTCGTTGATGGCAACA |
| iNOS | F：CGAAACGCTTCACTTCCAA  R：TGAGCCTATATTGCTGTGGCT |
| COX-2 | F：GATGCTCTTCCGAGCTGTG  R：GGATTGGAACAGCAAGGATTT |
| TNF-α | F：CTGTAGCCCACGTCGTAGC  R：TTGAGATCCATGCCGTTG |
| IL-6 | F：TCTAATTCATATCTTCAACCAAGAGG  R：TGGTCCTTAGCCACTCCTTC |
| IL-1β | F：TTGACGGAGCCCAAAAGAT  R：GATGTGCTGCTGCGAGATT |
| NF-κB | F：GGATGACAGAGGCGTGTATTAG  R：CCTTCTCTCTGTCTGTGAGTTG |

F:forward; R: reverse.

**Table S2 Information on 19 key aroma compounds with ROAV values greater than or equal to 10000 in four *Forsythia suspensa* fruit powders.**

| **Class** | **Compounds** | **CAS** | **Odor description** | **Threshold (mg·kg-1)** | **rOVAs** | | | |
| --- | --- | --- | --- | --- | --- | --- | --- | --- |
|  |  |  |  |  | **FS50** | **FS100** | **FS150** | **FS200** |
| Aldehyde | 2,6-Nonadienal, (E,Z)- | 557-48-2 | cucumber, green | 0.00001 | 4375941.17 | 43659.90 | 34471.11 | 1752529.79 |
|  | 2-Nonenal, (E)- | 18829-56-6 | fatty, green, cucumber, aldehydic, citrus | 0.00008 | 1776719.51 | 19685.23 | 17668.10 | 725877.26 |
|  | 2-Nonenal | 2463-53-8 | fatty, green, waxy, cucumber, melon | 0.0001 | 1421375.60 | 17748.19 | 16134.48 | 580701.81 |
|  | 2,6-Nonadienal, (E,E)- | 17587-33-6 | fresh, citrus, green, cucumber, melon | 0.0005 | 87518.82 | 10873.20 | 10689.42 | 35050.60 |
|  | Non-8-enal | 39770-04-2 | smoky, plastic | 0.0002 | 37496.87 | 146200.81 | 93100.96 | 13681.98 |
|  | 2-Nonenal, (Z)- | 60784-31-8 | orris, fatty, waxy, cucumber | 0.0045 | 31586.12 | 11172.18 | 11136.32 | 12904.48 |
| Alcohol | 2-Furfurylthiol | 98-02-2 | sulfury, roasted, coffee, oily, fatty, burnt, smoky | 0.000006 | 9037859.50 | 198485.49 | 161846.82 | 1161769.63 |
|  | 2-Thiophenemethanethiol | 6258-63-5 | roasted, coffee, fishy | 0.00004 | 106059.77 | 18430.74 | 15916.24 | 40320.72 |
| Ester | 3-Mercapto-3-methylbutyl formate (ester) | 50746-10-6 | sulfury, catty, caramel, onion, roasted coffee, roasted meat, tropical | 0.000002 | 875797.61 | 441292.06 | 459361.10 | 223844.53 |
|  | 2-Ethyl-n-butyric acid ethyl ester | 2983-38-2 | - | 0.000007 | 1195022.36 | 71690.00 | 64545.95 | 157023.22 |
| Ketone | 1-Octen-3-one | 4312-99-6 | mushroom | 0.000005 | 174104.09 | 164650.70 | 142789.74 | 496603.17 |
|  | 3-Octen-2-one | 1669-44-9 | earthy, spicy, herbal, sweet, mushroom, hay, blueberry | 0.00003 | 473267.05 | 141235.27 | 133404.45 | 119082.79 |
| Terpenoids | (2S,4R)-4-Methyl-2-(2-methylprop-1-en-1-yl)tetrahydro-2H-pyran | 3033-23-6 | rose, cortex, green, floral, geranium, powdery, metallic | 0.0002 | 47888.93 | 62389.68 | 40587.09 | 17999.37 |
|  | 2H-Pyran, tetrahydro-4-methyl-2-(2-methyl-1-propenyl)- | 16409-43-1 | sweet, floral, aromatic, rose, fresh, bay, leafy | 0.0002 | 47888.93 | 62389.68 | 40587.09 | 17999.37 |
| Sulfur compounds | Diethyl disulfide | 110-81-6 | gassy, ripe onion, greasy, garlic | 0.0000072 | 614339.68 | 710413.75 | 600219.41 | 77339.74 |
| Heterocyclic compound | Pyrazine, 2-methoxy-3-(2-methylpropyl)- | 24683-00-9 | green bell pepper, pea, galbanum | 0.000002 | 215856.16 | 25069816.00 | 20639573.49 | 66741.17 |
| Nitrogen compounds | Octanenitrile | 124-12-9 | fatty, aldehydic, green | 0.00013 | 39755.74 | 13240.10 | 12647.51 | 13730.91 |
| Aromatics | Benzene, (2-methylpropyl)- | 538-93-2 | - | 0.0008 | 53098.44 | 109821.62 | 107853.28 | 13128.48 |
| Hydrocarbons | (3E,5Z)-1,3,5-Undecatriene | 51447-08-6 | - | 0.00002 | 340530.67 | 527116.64 | 455614.30 | 144906.46 |


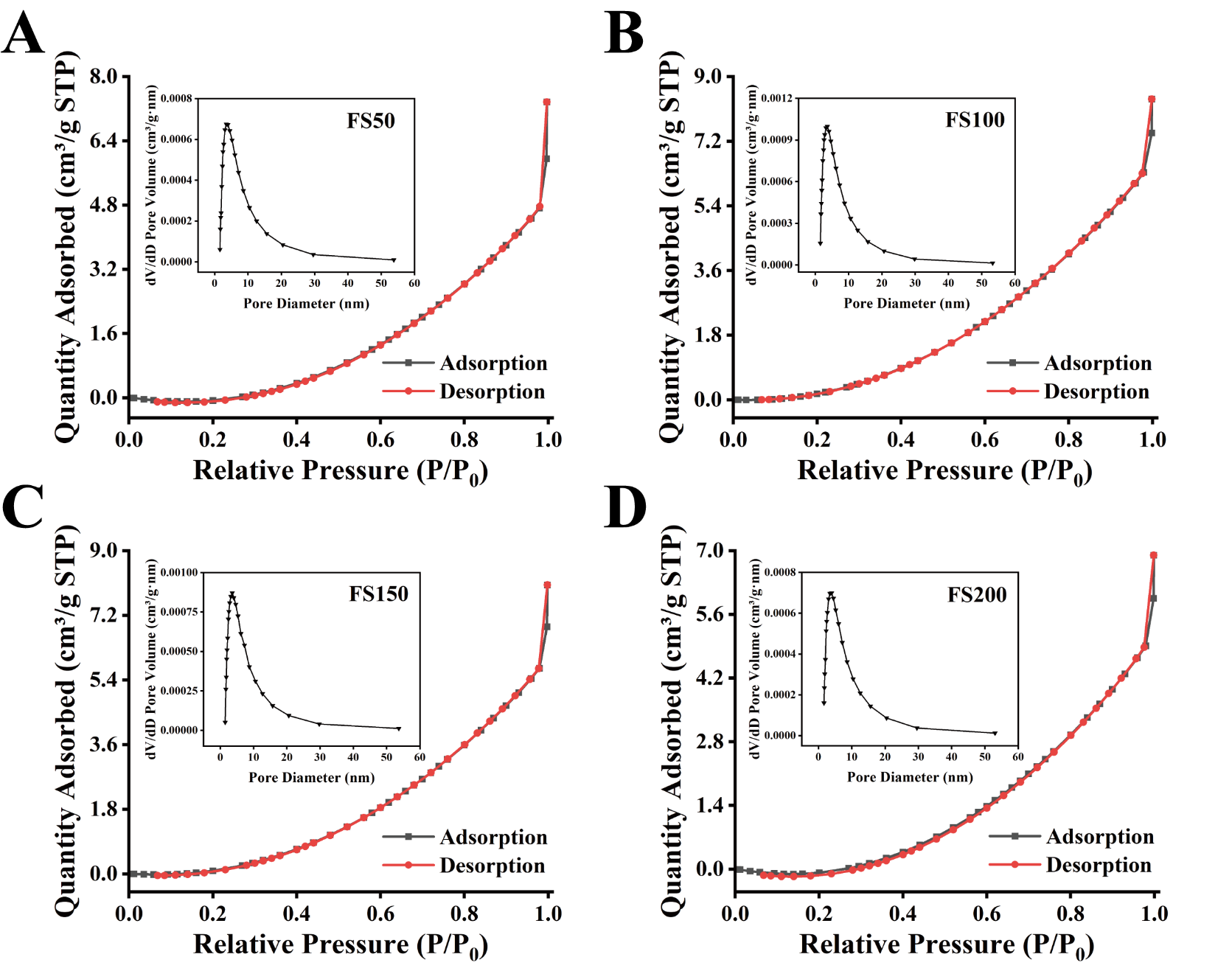


**Fig. S1. Nitrogen adsorption-desorption isotherms and pore size distribution curves of the *F. suspensa* fruit powders. (A) FS50; (B) FS100; (C) FS150; (D) FS200.**
